# A Case of Cerebral Arteriovenous Malformation and Malformation of the Lower Limbs

**DOI:** 10.7759/cureus.58336

**Published:** 2024-04-15

**Authors:** Tomohiro Nakajima, Tsuyoshi Shibata, Yutaka Iba, Keitaro Nakanishi, Nobuyoshi Kawaharada

**Affiliations:** 1 Cardiovascular Surgery, Sapporo Medical University, Sapporo, JPN

**Keywords:** embolism, bleeding, pro-glide, malformation of the popliteal artery, cerebral arteriovenous malformation

## Abstract

The case involves a 37-year-old female who was diagnosed with undifferentiated immunodeficiency and protein-losing gastroenteropathy at the age of 26 and was under outpatient care in the gastroenterology department while taking Prednisolone 15mg. At the age of 37, she experienced loss of consciousness and was diagnosed with a right occipital lobe arteriovenous malformation upon investigation. Although initially managed conservatively, she presented the following month with a right-sided headache and vomiting and was urgently transported to our hospital. Imaging with contrast-enhanced CT revealed bleeding from the arteriovenous malformation. Emergency craniotomy was performed, followed by ventricular drainage. Two weeks later, she underwent transcatheter arterial embolization of the main feeder via the right femoral artery approach, followed by excision of the arteriovenous malformation the next day. Subsequently, she had an uneventful recovery. A confirmation CT angiography before discharge revealed severe stenosis of the right common femoral artery, leading to a referral to the cardiovascular surgery department. The stenosis was attributed to the Pro-Glide used for hemostasis during the embolization procedure. Repair surgery was performed, during which CT angiography revealed arteriovenous malformations in both the popliteal fossae and the foot.

## Introduction

Cerebral arteriovenous malformations (AVMs) are reported to occur at a frequency of one in 100,000 individuals and can be a cause of cerebral hemorrhage in individuals aged 20 to 40 years [1]. The frequency of AVMs in the lower extremities is rare. Venous malformations are anomalies in vascular development during fetal life, characterized by the proliferation of venous-like vascular channels with low-flow blood circulation within subcutaneous tissues or muscles, forming dilated venous cavities due to developmental defects [2]. They can occur in any part or organ of the body, presenting with symptoms such as pain, swelling, functional impairment, and bleeding, either due to compression of surrounding tissues or thrombus formation, and can also pose cosmetic concerns. While they often manifest from birth due to their congenital nature, the onset of symptoms in adulthood is not uncommon. Management options include observation or conservative treatments (such as elastic stockings, anti-inflammatory analgesics, and antithrombotic agents) for asymptomatic or stable lesions, while symptomatic cases may require more aggressive interventions. In this case, a 37-year-old female diagnosed with cerebral AVM underwent treatment and was subsequently found to have AVMs in the bilateral lower legs and feet during the course of her treatment [3].

## Case presentation

**Figure 1:**
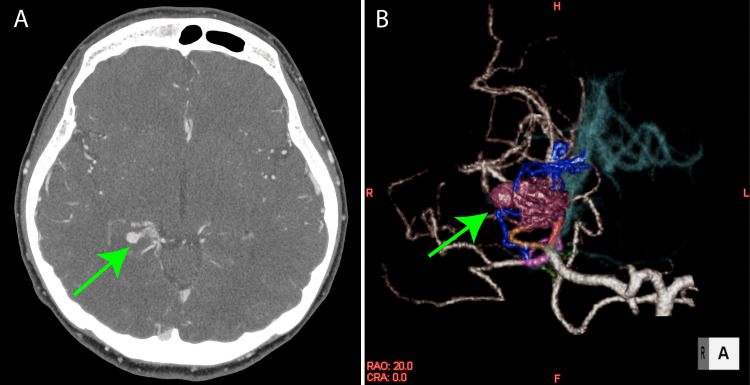
Preoperative images (A) Head-enhanced computed tomography. A horizontal axial view showed arteriovenous malformation at the left middle cerebral artery (green arrow). (B) Volume rendering image revealed arteriovenous malformation at the left middle cerebral artery (green arrow).

The case involves a 37-year-old female who was diagnosed with undifferentiated immunodeficiency syndrome and protein-losing gastroenteropathy at the age of 26. She was under the care of a gastroenterologist and was taking Prednisolone 15mg orally. At the age of 37, she experienced loss of consciousness, and further evaluation revealed a cerebral AVM in the right occipital lobe (Figures 1A, 1B).

**Figure 2:**
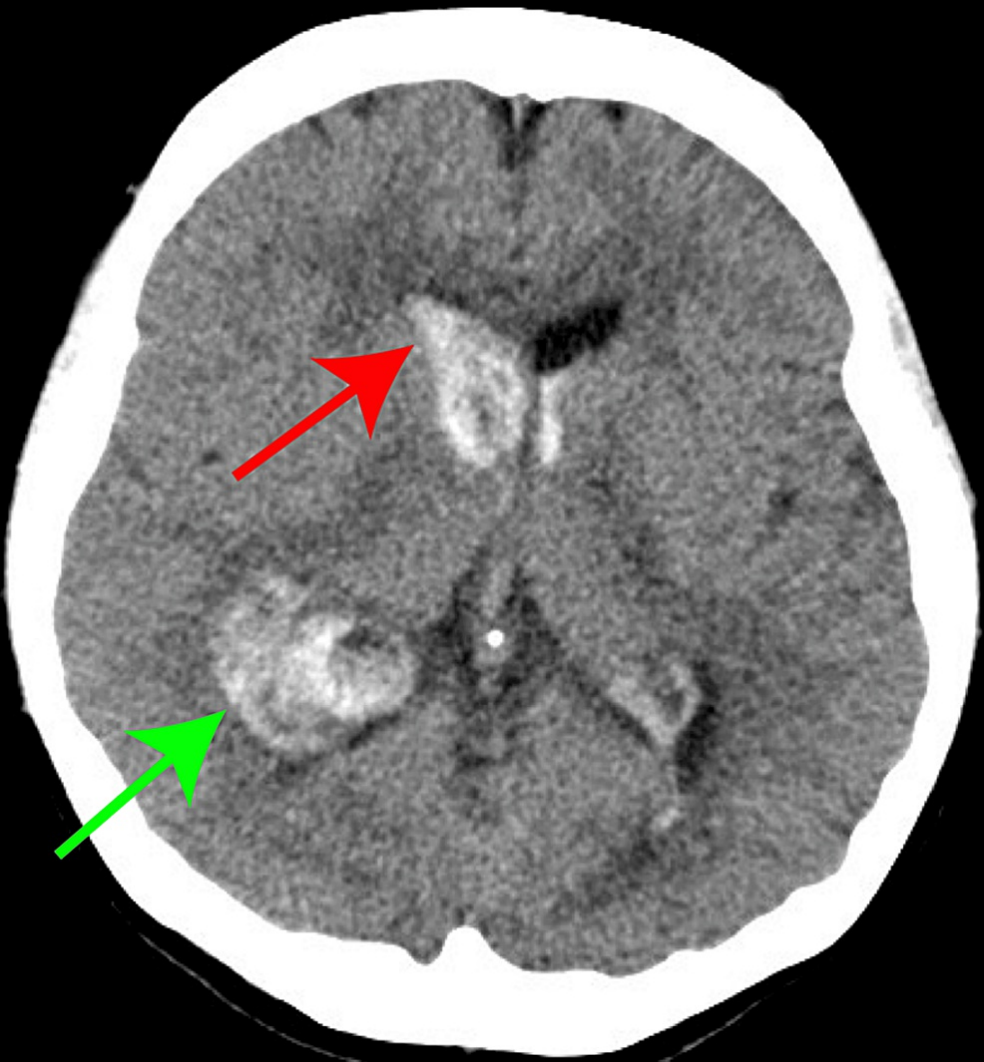
Computed tomography images Intracerebral hemorrhage in the occipital lobe was observed (green arrow), and intraventricular perforation was observed (red arrow).

Due to a low risk of rupture, she was placed under observation. The following month, she presented at home with right-sided headache and vomiting, leading to emergency transport to our hospital. Imaging with contrast-enhanced CT revealed bleeding from the AVM (Figure 2), necessitating emergency craniotomy and ventricular drainage.

**Figure 3:**
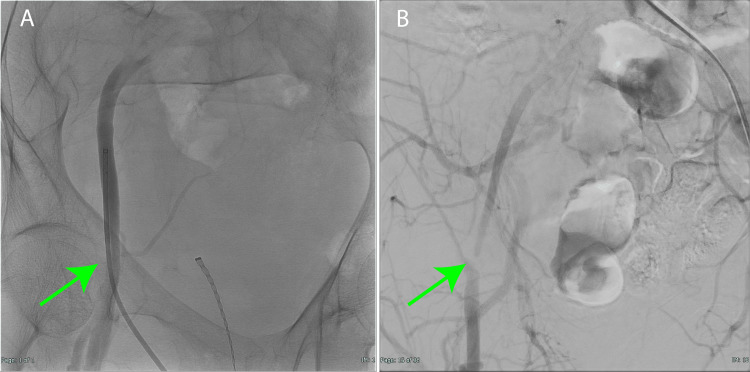
Angiography images (A) Angiography of right femoral artery puncture angiography for embolization; a sheath was inserted into the right common femoral artery, but there was no stenosis (green arrow). (B) Confirmatory angiography 14 days later showed severe stenosis of the right common femoral artery (green arrow).

Two weeks later, embolization of the main feeder to the AVM was performed via the right femoral artery approach, followed by excision of the cerebral AVM the next day. Subsequently, she had an uneventful recovery. A confirmatory contrast-enhanced imaging study before discharge revealed severe stenosis of the right common femoral artery (Figures 3A, 3B), prompting referral to the cardiovascular surgery department.

**Figure 4:**
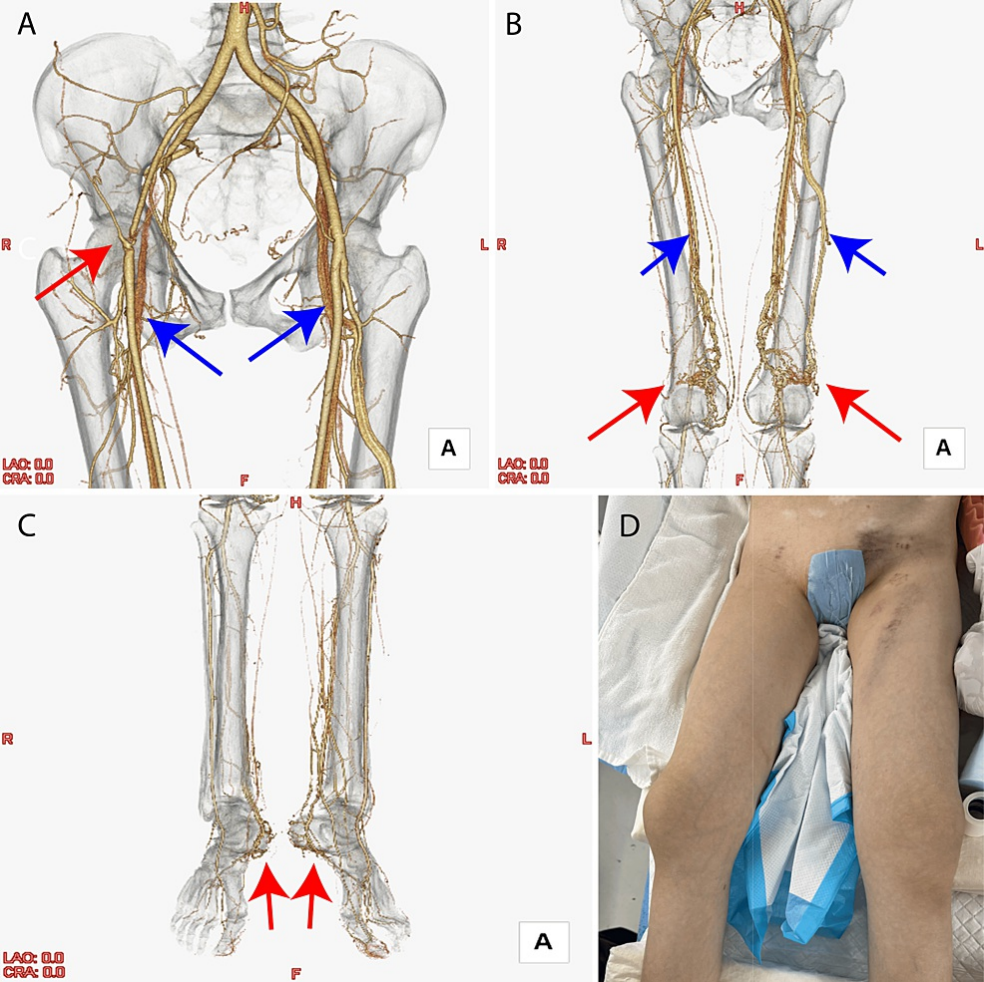
Computed tomography volume rendering images (A) Severe stenosis of the right common femoral artery (red arrow). This was an image of the arterial phase, but veins were depicted (blue arrow). (B) Images of bilateral thighs. Arteriovenous malformation was observed near the knee (red arrow), and veins were visible (blue arrow). (C) Bilateral arteriovenous malformations in the feet (red arrow). (D) Operative findings. There was no bilateral lower extremity vasoelasticity.

CT angiography demonstrated severe stenosis of the right common femoral artery, likely attributable to the Pro-Glide used for hemostasis during the embolization procedure. Additionally, imaging revealed cerebral AVMs in both popliteal fossae and the feet (Figures 4A-4D).

**Figure 5:**
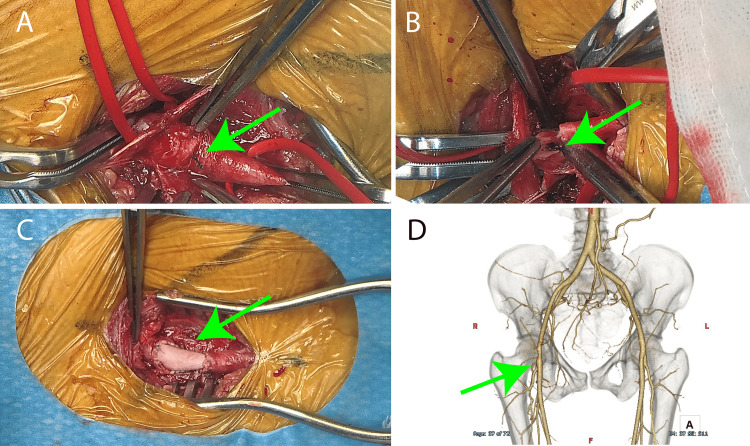
Intraoperative images and postoperative computed tomography (A) Intraoperative image. Pro-Glide knot was shown on the surface of the right femoral artery (green arrow). (B) Pro-Glide knot was sawn at the inner bottom of the right femoral artery (green arrow). (C) Right femoral artery plasty was conducted by patch (green arrow). (D) Postoperative image. Volume rendering computed tomography revealed the right femoral patch plasty (green arrow).

Under general anesthesia, repair of the right common femoral artery was performed (Figures 5A-5D).

Visual inspection revealed the Pro-Glide knot on the anterior wall of the right common femoral artery. Upon incision, the knot was found to be adhered to the posterior wall of the vessel. The Pro-Glide was secured to the posterior wall, resulting in stenosis, as it inadvertently compressed the vessel walls, including the anterior wall, during hemostasis. As the anterior wall of the right common femoral artery lacked an intact outer membrane, vascular reconstruction was performed using bovine pericardial patch. Despite being on Prednisolone, there were no issues with wound healing, and she was discharged home on postoperative day 9.

## Discussion

Cerebral AVM is a vascular disease characterized by abnormal connections between arteries and veins in the brain, forming a tangled mass of vessels [4]. Due to their thinner walls compared to normal blood vessels, AVMs are prone to rupture, leading to cerebral hemorrhage or subarachnoid hemorrhage. It is often detected in individuals aged 20 to 40, making it a known cause of cerebral hemorrhage in young adults. Symptoms such as headaches and seizures may occur even without bleeding [5]. Some individuals may remain asymptomatic, with the condition being incidentally discovered during brain imaging. AVMs can occur anywhere in the brain and often do not cause symptoms solely due to their presence. They are typically detected either when bleeding occurs, or incidentally during brain imaging. Other presentations may include headaches or seizures. In this case, the discovery of the cerebral AVM was prompted by loss of consciousness, and it subsequently ruptured, leading to cerebral hemorrhage. The patient underwent cranial hematoma drainage for cerebral hemorrhage and completed the acute stage treatment. Two weeks later, the feeding artery to the AVM was embolized, and the next day, the cranium was opened and the AVM was removed. Pro-Glide was used for hemostasis following catheter treatment. According to Xiang et al., complications associated with Pro-Glide have been reported at a frequency of 1.0%-1.7% [6]. In this case, as the right common femoral artery was not problematic before the cerebral AVM embolization, the complication was attributed to Pro-Glide. Surgical findings revealed the presence of the Pro-Glide knot on the posterior wall of the right common femoral artery. It was presumed to have occurred during Pro-Glide deployment, lifting and securing the posterior wall of the femoral artery. Arterial reconstruction was performed using a XenoSure patch, with no subsequent issues. Incidentally, AVMs were discovered in the popliteal fossae and feet. While detailed reports on the frequency of lower limb AVMs are lacking, Akita et al. reported that their occurrence is about half that of cerebral AVMs [1]. Cases requiring treatment for lower limb AVMs typically involve high-flow lesions or lesions causing skin ulcers. However, in this case, neither condition was present, leading to observation without intervention [7].

## Conclusions

A patient who experienced bleeding due to cerebral AVM developed severe stenosis of the right common femoral artery during the course of her embolization treatment. The stenosis was severe enough to necessitate surgical repair. During the evaluation of her lower limbs, AVMs were incidentally discovered in both popliteal fossae and feet. As the AVMs were asymptomatic and not high-flow lesions, they were managed with observation. This case highlights an instance where a woman with symptomatic cerebral AVM also had AVMs incidentally found in both popliteal fossae and feet during lower limb evaluation.
